# La Crosse Virus in *Aedes albopictus* Mosquitoes, Texas, USA, 2009

**DOI:** 10.3201/eid1605.100170

**Published:** 2010-05

**Authors:** Amy J. Lambert, Carol D. Blair, Mary D’Anton, Winnann Ewing, Michelle Harborth, Robyn Seiferth, Jeannie Xiang, Robert S. Lanciotti

**Affiliations:** Centers for Disease Control and Prevention, Fort Collins, Colorado, USA (A.J. Lambert, R.S. Lanciotti); Colorado State University, Fort Collins (C.D. Blair); Texas Department of State Health Services, Austin, Texas, USA (M. D’Anton, W. Ewing, M. Harborth, R. Seiferth, J. Xiang)

**Keywords:** Viruses, vector-borne, arbovirus, Texas, encephalitis, mosquitoes, dispatch

## Abstract

We report the arthropod-borne pediatric encephalitic agent La Crosse virus in *Aedes albopictus* mosquitoes collected in Dallas County, Texas, USA, in August 2009. The presence of this virus in an invasive vector species within a region that lies outside the virus’s historically recognized geographic range is of public health concern.

La Crosse virus (LACV) is the most common cause of arthropod-borne, pediatric encephalitis in North America. A member of the California serogroup within the family *Bunyaviridae* and the genus *Orthobunyavirus,* LACV is enveloped and contains a negative-sense, tripartite genome with segments designated small (S), medium (M), and large (L). Cases of LACV-associated encephalitis, which can be fatal, occur within the geographic range of its principal vector, *Aedes triseriatus* mosquitoes. This native tree-hole breeding mosquito is distributed throughout wooded regions east of the Rocky Mountains within the United States. Historically, most LACV-associated encephalitis cases have occurred in upper midwestern states, including Wisconsin, Illinois, Minnesota, Indiana, and Ohio (Figure 1). In recent years, LACV encephalitis activity has increased above endemic levels in regions of the southeastern United States, including West Virginia, North Carolina, and Tennessee (Figure 1) (*1*). In addition, recent cases of LACV encephalitis have been reported as far south as Louisiana, Alabama, Georgia, and Florida (Figure 1).

**Figure 1:**
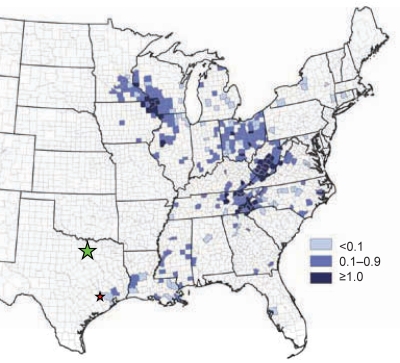
Geographic distribution of La Crosse virus (LACV) in accordance with the habitat range of *Aedes triseriatus* mosquitoes in the United States as inferred from the California serogroup virus neuroinvasive disease average annual incidence by county, 1996–2008. Incidence rates are shown in shades of blue. Dallas County and Fort Bend County locations of the 2009 LACV isolations from pools containing *Ae.albopictus* and *Ae. triseriatus* mosquitoes are indicated by green and red stars, respectively. Data and figure adapted from the Centers for Disease Control and Prevention website (www.cdc.gov/lac/tech/epi.html).

*Ae. albopictus* is an invasive mosquito species that was first discovered in Houston, Texas, in 1985 (*2*); the mosquitoes apparently arrived in the United States in a shipment of used tires from Asia (*3*). An opportunistic container-breeder, its vector competence for many arthropod-borne viruses (arboviruses), including LACV, and its catholic feeding habits have made the invasion of *Ae. albopictus* mosquitoes disconcerting to researchers, who have warned of the potential for an increased incidence of vector-borne diseases as a result (*4*,*5*).

Since 1985, the geographic distribution of these mosquitoes has grown to include most of the southeastern United States. The concurrent increase in LACV encephalitis activity has led to speculation on the possible transmission of LACV by *Ae. albopictus* mosquitoes as an accessory mechanism to the historically recognized transmission by *Ae. triseriatus* mosquitoes (*6*). LACV has been isolated from *Ae. albopictus* mosquitoes in Tennessee and North Carolina in 1999 and 2000, respectively, during a period of greatly increased LACV activity in those areas (*6*). However, the role of this species in LACV transmission remains unknown.

We report the isolation of LACV from a pool of 3 *Ae. albopictus* mosquitoes collected outside the known geographic range of the virus, in Dallas County, Texas, on August 13, 2009 (Figure 1). This is one of only several isolations of LACV within the state; the first isolate was derived from a pool of *Ae. infirmatus* mosquitoes collected in the Houston in 1970 (*7*). After the identification of LACV in the Dallas pool, an additional isolation of LACV was made from a mixed pool of 29 *Ae. albopictus* and 2 *Ae. triseriatus* mosquitoes collected in Fort Bend County, Texas, in October 2009 (Figure 1). The Fort Bend County location is relatively near the site of collection of the 1970 Texas LACV-positive pool and the known geographic distribution of LACV activity in southeastern Texas and Louisiana (Figure 1). Taken together, our results represent an unprecedented number of LACV findings within the state of Texas.

## The Study

As part of ongoing arbovirus surveillance efforts, the City of Dallas Vector Control Division collected 65 mosquitoes in a gravid trap at the edge of a wooded area near a residential district in Dallas County on August 13, 2009. Upon their receipt at the Texas State Department of Health Services, none of the mosquitoes was viable. The mosquitoes were sorted and identified by sex. Female mosquitoes were grouped into 3 pools by species: pool no. AR6318, consisting of 50 *Culex quinquefasciatus* mosquitoes, pool no. AR6319, consisting of 3 *Ae. albopictus* mosquitoes; and pool no. AR6320, consisting of 1 *Ae. triseriatus* mosquito.

Generated pools were macerated in 1.5 mL of bovine albumin diluent arbovirus medium followed by 2 rounds of centrifugation at 10,000 rpm for 5 min each. Between each round of centrifugation, a rest period of 15 min was used to facilitate pellet formation. After centrifugation, 50 µL of the resultant supernatant was injected onto BHK and Vero cells. These cells were incubated at 37°C and examined for cytopathic effect (CPE) over the next 10 days. At day 5 postinoculation, Vero cells inoculated with the supernatant derived from pool no. AR6319 (*Ae. albopictus*) demonstrated marked CPE. This condition represented a preliminary virus isolation-positive result. No CPE was observed in the BHK cells. Infected cells were then subjected to immunofluorescent antibody assays with antibodies directed against various arboviruses, followed by the use of fluorescein isothiocyanate–conjugated antimouse antibodies for detection. From these analyses, the isolate derived from pool no. AR6319 (*Ae. albopictus*) was determined to be a California serogroup virus. Furthermore, pool no. 6318 (*Cx. quinquefasciatus*) tested positive for West Nile virus, and pool no. 6320 (*Ae. triseriatus*) was negative for virus by the above described methods.

To further identify the California serogroup virus identified in pool no. AR6319 (*Ae. albopictus*), the pool and the Vero cell–derived isolate were sent to the Centers for Disease Control and Prevention in Fort Collins, CO, USA, for additional testing. Upon receipt of the samples at Fort Collins, a reverse transcription–PCR was performed to amplify cDNAs from all 3 segments of the orthobunyavirus genome by using the consensus oligonucleotide primers shown in the Table and conditions and methods previously described (*8*). Generated cDNAs were then subjected to nucleotide sequencing and BLAST (*www.ncbi.nlm.nih.gov/BLAST)* analyses; the results indicated that the pool and the isolate were positive for LACV S, M, and L segment RNAs.

**Table:** Orthobunyavirus consensus oligonucleotide primers used for amplification and sequencing of La Crosse virus partial S, M, and L segment cDNAs, Texas, 2009*

Targeted genomic regions	Name	Primer sequence (5′ → 3′)	Approximate amplicon size, bp
S segment nucleocapsid ORF	Cal S forward	GCAAATGGATTTGATCCTGATGCAG	210
	Cal S reverse	TTGTTCCTGTTTGCTGGAAAATGAT	
M segment 5′ terminus/glycoprotein ORF	Ortho M 5′ terminus	AGTAGTGTACTACC	410
	Ortho M ORF reverse	TTRAARCADGCATGGAA	
L segment 5′ terminus/polymerase ORF	Ortho L 5′ terminus	AGTAGTGTACTCCTA	550
	Ortho L ORF reverse	AATTCYTCATCATCA	

*Oligonucleotide primers designed against conserved regions of the orthobunyavirus genome. S segment primers appear in a previous publication (*9*). All primers were applied in singleplex reactions using methods described previously (*9*) with altered primer annealing conditions of 50^o^C for 1 min. S, small; M, medium; L, large; ORF, open reading frame.

Subsequently, a pool (AR8973) of 29 *Ae. albopictus* and 2 *Ae. triseriatus* mosquitoes collected in Fort Bend County, Texas on October 5, 2009, was identified as positive for LACV S, M, and L segment RNAs by using the same processing and characterization methods described above. After these analyses, full-length S, M, and L segment genomic sequences (GenBank accession nos. GU591164–9) were generated for LACV RNAs extracted from LACV-positive pools and Vero cell isolates by using oligonucleotide primers specific for the previously published LACV prototype genome (human 1960, GenBank accession nos. EF485030–2) and methods previously described (*9*).

**Figure 2:**
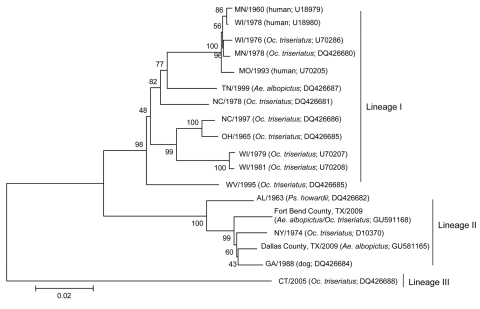
Phylogeny of La Crosse virus (LACV) medium (M) segment sequences of diverse origins. According to a limited availability of full-length sequences in GenBank, 1,663 nt of the M segment glycoprotein gene open-reading frame are compared. Isolate source and GenBank accession nos. appear after the isolate designation for each taxon. Sequences were aligned by ClustalW (*10*) and neighbor-joining and maximum-parsimony trees were generated by using 2,000 bootstrap replicates with MEGA version 4 software (*10*). Highly similar topologies and confidence values were derived by all methods and a neighbor-joining tree is shown. Scale bar represents the number of nucleotide substitutions per site. The 2009 Texas (TX) isolates group with strong support with lineage 2 viruses of the extreme south and New York (NY), which suggests a likely southern origin for LACV isolates. MN, Minnesota; WI, Wisconsin; *Oc*., *Ochlerotatus*; MO, Missouri; TN, Tennessee; *Ae*., *Aedes*; NC, North Carolina; OH, Ohio; WV, West Virginia; AL, Alabama; *Ps*., *psorophora*; GA, Georgia; CT, Connecticut.

Phylogenetic analyses of partial LACV M segment sequences (Figure 2) indicate that the LACVs present in the Texas 2009 pools are closely related to LACVs isolated from Alabama, Georgia, and New York of the previously described lineage 2 (*11*) and genotype C (*7*) designations. These findings suggest a likely southeastern ancestry for the Texas 2009 LACV isolates.

## Conclusions

The presence of LACV in *Ae. albopictus* mosquitoes in Dallas County, Texas, in late summer 2009 represents the possible expansion of the geographic range of an endemic pathogen within this invasive mosquito species in the United States. The subsequent occurrence of LACV in Fort Bend County in October 2009 should be of concern to public health practitioners who have been alerted to the presence of this pathogen near 2 major urban centers, Dallas and Houston. Of interest, San Angelo virus, which is serologically related to LACV, is known to occur in Texas and has been shown to replicate in and be transovarially transmitted by *Ae. albopictus* mosquitoes (*12*), although this virus has no known association with human disease. Cocirculation enables possible reassortment of genomic segments between LACV and San Angelo virus, a phenomenon that has been described for viruses of the California serogroup within *Ae. albopictus* mosquitoes (*13*) with unknown public health outcomes.

## References

[1] Haddow AD, Odoi A. The incidence risk, clustering, and clinical presentation of La Crosse virus infections in the eastern United States, 2003–2007. PLoS ONE. 2009;4:e6145 . 10.1371/journal.pone.0006145 19582158 PMC2702082

[2] Centers for Disease Control and Prevention. *Aedes albopictus* introduction—Texas. MMWR Morb Mortal Wkly Rep. 1986;35:141–2. 3080671

[3] Moore CG, Mitchell CJ. *Aedes albopictus* in the United States: ten-year presence and public health implications. Emerg Infect Dis. 1997;3:329–34. 10.3201/eid0303.970309 9284377 PMC2627635

[4] Grimstad PR, Kobayashi JF, Zhang MB, Craig GB Jr. Recently introduced *Aedes albopictus* in the United States: potential vector of La Crosse virus (*Bunyaviridae:* California serogroup). J Am Mosq Control Assoc. 1989;5:422–7. 2584976

[5] Weaver SC, Reisen WK. Present and future arboviral threats. Antiviral Res. 2010;85:328–45. Epub 2009 Oct 24. 10.1016/j.antiviral.2009.10.008 19857523 PMC2815176

[6] Gerhardt RR, Gottfried KL, Apperson CS, Davis BS, Erwin PC, Smith AB, First isolation of La Crosse virus from naturally infected *Aedes albopictus.* Emerg Infect Dis. 2001;7:807–11. 10.3201/eid0705.010506 11747692 PMC2631884

[7] Klimas RA, Thompson WH, Calisher CH, Clark GC, Grimstad PR, Bishop DH. Genotypic varieties of La Crosse virus isolated from different geographic regions of the continental United States and evidence for a naturally occurring intertypic recombination of La Crosse virus. Am J Epidemiol. 1981;114:112–31. 7246519 10.1093/oxfordjournals.aje.a113158

[8] Lambert AJ, Lanciotti RS. Consensus amplification and novel multiplex sequencing method for S segment species identification of 47 viruses of the *Orthobunyavirus, Phlebovirus*, and *Nairovirus* genera of the family *Bunyaviridae.* J Clin Microbiol. 2009;47:2398–404. 10.1128/JCM.00182-09 19535518 PMC2725646

[9] Lambert AJ, Lanciotti RS. Molecular characterization of medically important viruses of the genus *Orthobunyavirus.* J Gen Virol. 2008;89:2580–5. 10.1099/vir.0.2008/002253-0 18796727

[10] Tamura K, Dudley J, Nei M, Kumar S. MEGA4: Molecular Evolutionary Genetics Analysis (MEGA) software version 4.0. Mol Biol Evol. 2007; 1596–9. Epub 2007 May 7. 10.1093/molbev/msm092 17488738

[11] Armstrong PM, Andreadis TG. A new genetic variant of La Crosse virus *(Bunyaviridae)* isolated from New England. Am J Trop Med Hyg. 2006;75:491–6. 16968927

[12] Tesh RB, Shroyer DA. The mechanism of arbovirus transovarial transmission in mosquitoes: San Angelo virus in *Aedes albopictus.* Am J Trop Med Hyg. 1980;29:1394–404. 7446826 10.4269/ajtmh.1980.29.1394

[13] Cheng LL, Rodas JD, Schultz KT, Christensen BM, Yuill TM, Israel BA. Potential for evolution of California serogroup bunyaviruses by genome reassortment in *Aedes albopictus.* Am J Trop Med Hyg. 1999;60:430–8. 10466972 10.4269/ajtmh.1999.60.430

